# Half a century of the reverse transcriptase—happy birthday!

**DOI:** 10.1186/s13059-020-02219-5

**Published:** 2021-01-11

**Authors:** Karin Moelling

**Affiliations:** 1 Institute of Medical Microbiology, University of Zurich, 8006 Zürich, Switzerland; 2 Max Planck-Institute of Molecular Genetics, 14195 Berlin, Germany

**Fig. 1:**
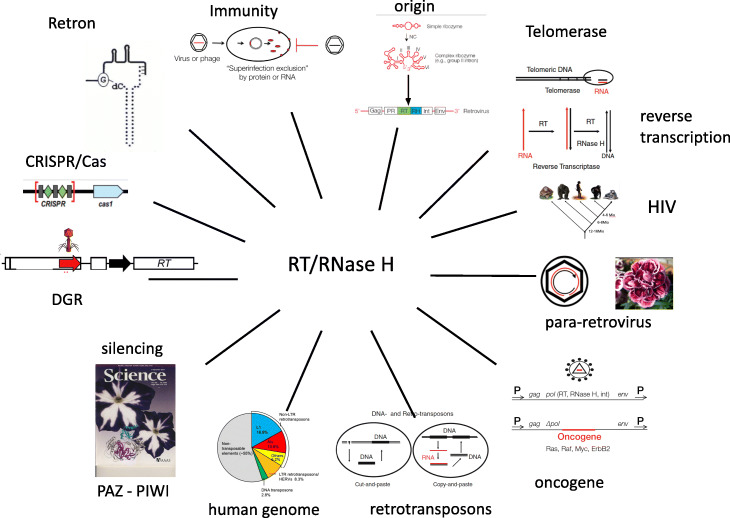
Some of the many facets of the reverse transcriptas (RT). For details, see text

The reverse transcriptase (RT) was discovered 50 years ago by Howard M. Temin, Satoshi Mitzutani [1], and David Baltimore [2]. A birthday party will not take place because of the SARS-coronavirus-2 pandemic. A meeting in Cold Spring Harbor (CSH) to celebrate this event with RT scientists was postponed by a year. However, this pandemic reminds us almost daily of the RT and makes it presently one of the most important molecules, because it is required to reverse transcribe the coronaviral RNA into DNA as a key step in the RT-qPCR test used to detect potential infections. Thus, the 50th birthday of the RT deserves congratulations and a retrospective about its contribution to our knowledge, our past, and a glance into the future summarized in Fig. 1.

The RT was thought first to be a rare exception in biology and restricted to retroviruses only, such as leukemia viruses of chickens and mice. The name retrovirus is based on the RT, allowing the unexpected flow of genetic information from RNA to DNA, which was considered to be reversed when it was discovered in 1970. Before that, information transfer was thought to only occur from DNA to RNA to protein as described by the Central Dogma of molecular biology coined by Sir Francis Crick—even though he was not dogmatic about it [3]. David Baltimore was surprised in the 1970s during a meeting when a speaker described an RT in flies—arguing that no retrovirus was known to exist there. That was the beginning of a ubiquitous RT. Now with so much emphasis on the RNA world, one could rename the RT as the real transcriptase, which nobody will do!

Retroviruses attracted attention for cancer research since they can pick up oncogenes and become tumor viruses. Retroviruses have many different vertebrate hosts with specific viruses in chickens, mice, sheep, goats, horses and so on, mainly causing leukemias and lymphomas, but they can also cause anemias and arthritis. To find a human retrovirus was the focus of intense research and international competition. Mason Pfizer Monkey virus and other proposals turned out to be wrong. When Luc Montagnier from the Institute Pasteur in Paris finally presented a human isolate in 1983, what would later be called HIV, more than one person in the CSH auditorium did not believe it [4]. But they were wrong to doubt. R.C. Gallo had discovered the first human tumor virus, HTLV-1, in 1980 [5].

HIV, of course, caused another pandemic, which is much neglected or overshadowed by the outbreak of the SARS-coronavirus-2 pandemic. HIV is a retrovirus, discovered in 1983, and replicates via the RT—and again, the RT is the basis for diagnostics and was the first target of a drug, AZT, in 1987.

RNA tumor viruses can carry oncogenes or activate downstream cellular genes by the viral promotors, the long terminal repeats (LTRs), by promoter insertion. The myc gene plays a role in both of the two mechanisms, causing an acute or chronic disease in animals [6], and is now being explored as a drug target in human cancer. The first prominent tumor retrovirus was the Rous sarcoma virus, RSV, which can replicate and carry an oncogene v-src. The question was the following: are oncogenes from outside of a cell or from within? A *Nature* preprint from Mike Bishop and coworkers in San Francisco was going around in 1971 or so at the Max Planck Institute of Virology in Tübingen, describing an “oncogene” present everywhere, even in elephants—with no correlation to cancer. The answer to this puzzle was that the oncogene looked the same as cellular genes, the “protooncogenes”, with the technologies available. But it was mutated in cancer, not identical. Mike Bishop and Harold Varmus received the Noble Prize for their discovery of oncogenes in 1989.

Harold Varmus attended European Tumor Virus Meetings on oncogenes by riding his bicycle from London across half of Europe even in heavy rain, a pioneer not only in cancer research but for bicycle riding 50 years before this becomes a need now by fear of SARS-coronavirus-2 infections in public transportations.

Many years back, around the 1970s, a slide was projected during one of the CSH meetings showing a healthy chicken which harbored an endogenous retrovirus. This was a surprise, because everybody was expecting a sick chicken, but this one looked perfectly normal. Endogenous viruses were also found in mice and were later identified as endogenous viruses in many species. This culminated in the publication of the most spectacular paper involving retroviruses—the human genome sequence by Eric Lander and numerous other authors and groups [7]. This revealed that retroviruses and retrovirus-like elements populate the human and other mammalian genomes to almost 50% even today, and in some genomes up to 80% [8]. This was proved by the isolation of “Phoenix,” a retrovirus reconstructed from the human genome from a dozen mutated sequences, which allowed the deduction of a consensus ancestral sequence. A DNA copy, transfected into tissue culture cells, produced the replicating retrovirus Phoenix, detectable by electron microscopy. After 5 million years of dormancy, the virus was brought back to “life” [9]. We harbor sequences of the virus Phoenix in our genome from former infections, but nobody seemed to worry about its recent resurrection in the laboratory as a potential danger!

A retrovirus contributed important novel information for the benefit of human evolution: preventing mothers from immune rejection of their own developing embryos. Egg shells and kangaroo pouches became obsolete by a piece of DNA coding for a region of the envelope protein of an ancestor of the human endogenous retrovirus, HERV-W [10]. The mechanism is closely related to immune suppression by AIDS and based on a peptide sequence also found in the gp41 fusion protein of HIV. Cells in the placenta of ancestors of humans and several other female mammals were infected independently 25 to 40 million years ago.

Endogenous viruses can protect against exogenous viruses. This immunity was shown in a real-time endogenization process observed with koalas in Australia. They were threatened by factors such as car accidents or fires and were put into custody to allow the population to recover on an island. It was not as safe a place as expected, because the Gibbon ape leukemia virus habited there and killed many of the koalas. Some of them survived and were immune because the virus had entered their germlines. This allowed scientists to watch such an evolutionary event of endogenization in real time [11]. Endogenization in the koalas took 100 years, about 10 generations. How long will HIV need to endogenize into human germ cell genomes and defend their offspring against superinfection? Infection seems possible and thus endogenization also? Ten koala generations may correspond to 250 years for humans—this is too long to wait for!

This protection by endogenous viruses against exogenous viruses is not dissimilar to one approach bacteria have evolved as defense against phages. A DNA fragment of an invading bacterial virus/phage is stored as memory, as a “spacer” within palindromic sequences. Its RNA transcript recognizes the DNA genome of an invading new phage by sequence homology and leads to its destruction by molecular scissors, the CRISPR/Cas effect [12], which everyone is aware won the Noble Prize for chemistry this year.

Bacteria harbor many RTs from retroelements. One of these RTs is highly mutagenic and drives evolution by promoting hypervariation of protein sequences, a property shared with the adaptive immune response. This leads to an expanded tropism of bacterial and phage interaction, possibly to adapt to environmental stress. The sequences carrying this RT are called DGR, diversity-generating retroelements [13]. They may help to create broad-spectrum phages. Another puzzling RT in bacteria belongs to retrons, which consist of branched single-stranded RNA/DNA structures [14] which Howard Temin had already noticed [15]. It looks like a conserved evolutionary intermediate from RNA to DNA. Bacterial RTs can also bind to catalytic RNAs (group II introns) [16]. They also resemble transition intermediates from the RNA to the DNA world with the RT as link between the two worlds [17] making the RT a major driver in evolution.

There is another relative of the RT. Also connected to the CSH Laboratory is TERT, the telomeric RT, described by Elizabeth Blackburn and Carol Greider, frequent guests at CSH, who received the Nobel prize together with Jack Szostak in 2009. TERT elongates chromosomal ends in embryonic tissue and stem cells and is active in cancer cells corresponding to longevity [18]. Unlike other RTs, an RNase H is not involved, since RNA is needed as a template for the repeated copies of the telomeric DNA. Linear chromosomes need protection of their ends against shortening which is provided by TERT and its stable pseudoknot RNA structure. TERT has been proposed to be related to non-LTR retrotransposons, which lost an endonuclease [19]. Is this the oldest ancestral RT precursor, a ribonucleoprotein consisting of non-coding pseudoknot-structured RNA and the RT enzyme or are introns the origin?

Last not least we give our adoration to Barbara McClintock for her discovery of mobile genetic elements, which include retrotransposons with their copy-and-paste mechanism. Her legacy will continue to keep researchers busy studying this prominent force of genomic evolution and adaptive processes and contribute to many more CSH meetings to come.

This remembrance has shown how broad the RTs are in nature and during evolution and how many open questions still wait for further analysis.

A bunch of flowers makes a nice birthday surprise, so at the end of this overview, we discuss two phenomena visible in plants, related to RT. The first is the white pattern of petunias, which is caused by silencing of the gene responsible for the blue coloring. The silencing depends on the PAZ and PIWI domains of Argonaute proteins which are structurally related to the retroviral RT and RNase H enzymes. They are components of the RNA-induced silencing complex RISC, which is believed to have evolved as an antiviral immune defense. In contrast, the white rims around carnation petals are caused by viruses, whereby one of them is the viroid of carnations, CarSVd (Carnation Small Viroid), and the other one a plant pararetrovirus. Its RT helps to make a homologous DNA from the viroid, allowing DNA recombination [20]. Thus, this viroid exploits an RT, presumably provided *in trans by* a plant pararetrovirus, such as cauliflower mosaic virus leading to color patterns.

**Fig. 2:**
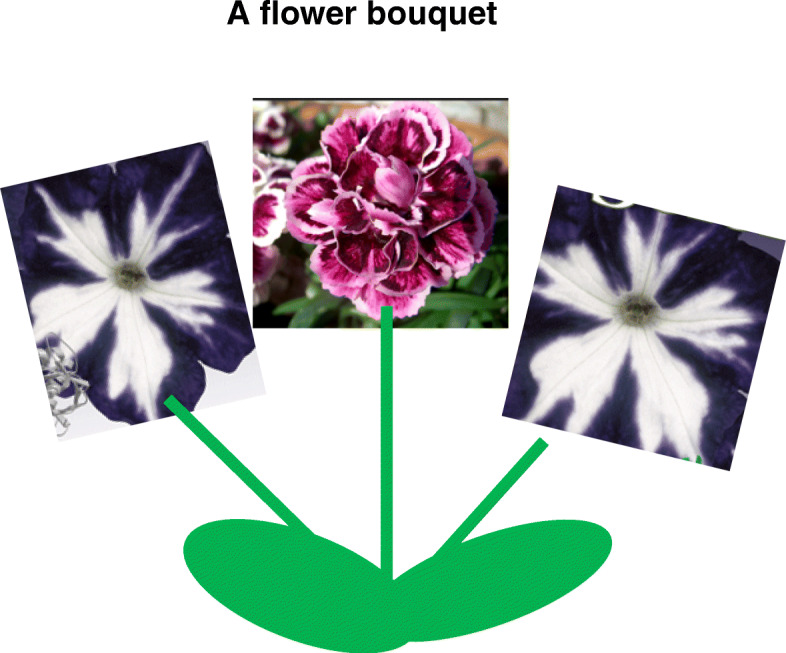
A flower bouquet

A flower bouquet with silenced petunias and multicolored carnations is a greeting for the birthday of the RT, whereby the beauty of flowers is due to RTs (Fig. 2).

Congratulations to David Baltimore and a remembrance to Howard Temin.
